# Supplementary figures and images for: Temporal origin of mouse claustrum and development of its cortical projections
Source: Cereb Cortex. 2022 Sep 14;33(7):3944–59. doi: 10.1093/cercor/bhac318 (PMC10068282; doi:10.1093/cercor/bhac318)

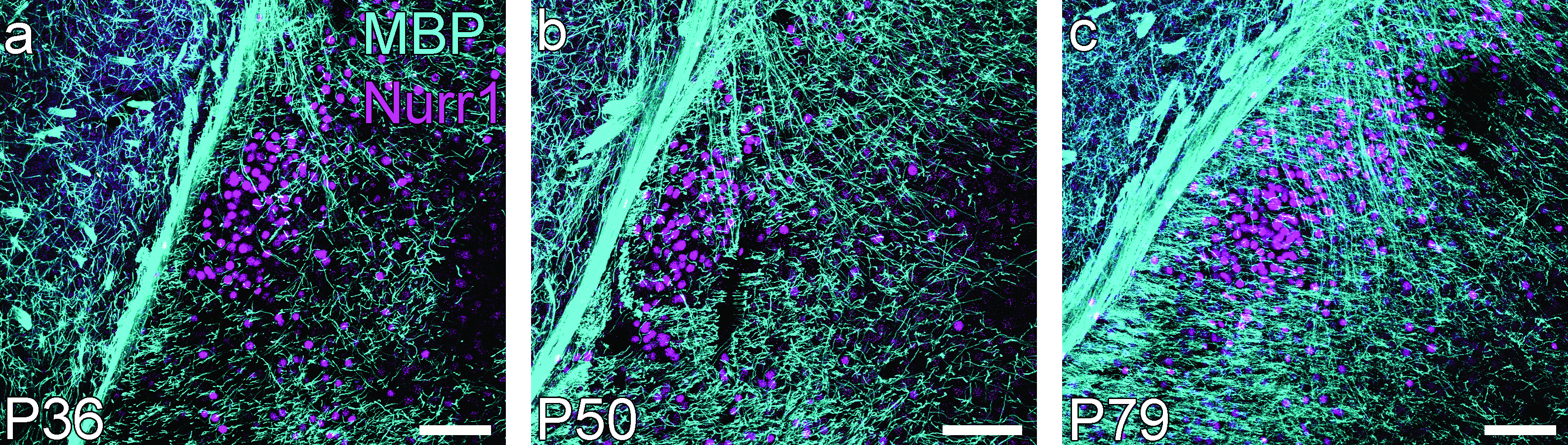

Supplement: Suppl_Fig_1_colour_bhac318 [file suppl_fig_1_colour_bhac318.zip › Suppl_Fig_1_colour_bhac318.tif]

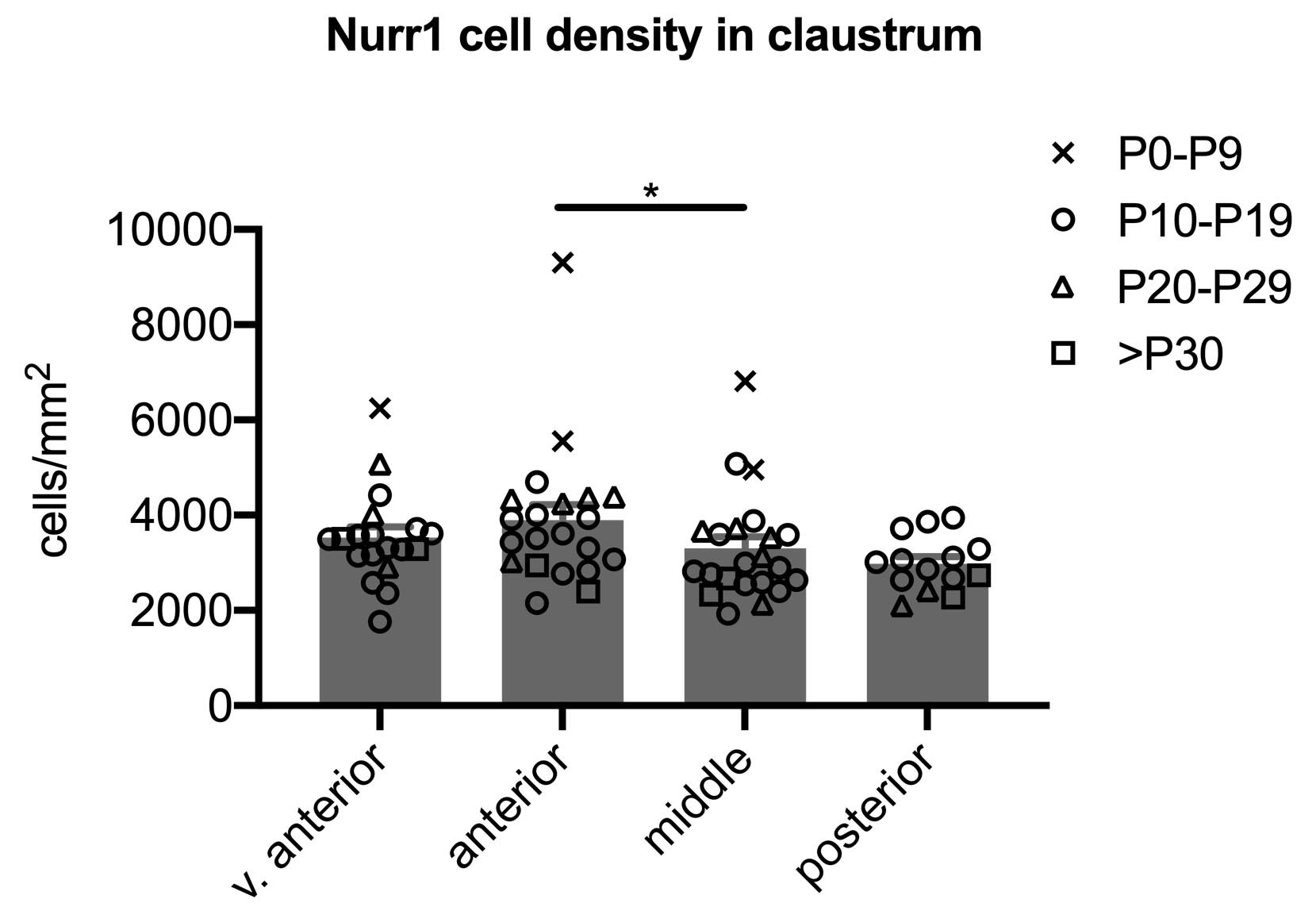

Supplement: Suppl_Fig_2_bhac318 [file suppl_fig_2_bhac318.jpeg]

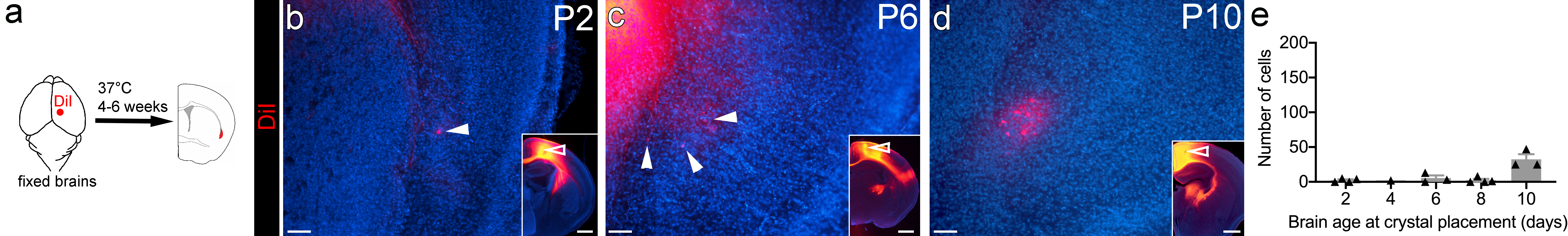

Supplement: Suppl_Fig3_colour_bhac318 [file suppl_fig3_colour_bhac318.zip › Suppl_Fig3_colour_bhac318.tif]

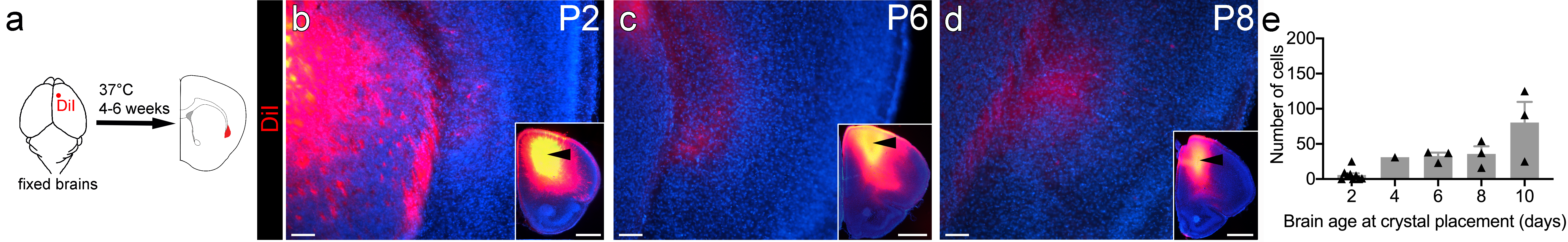

Supplement: Suppl_Fig4_colour_bhac318 [file suppl_fig4_colour_bhac318.zip › Suppl_Fig4_colour_bhac318.tif]
