# Supplementary material for: Temporal origin of mouse claustrum and development of its cortical projections
Source: Cereb Cortex. 2022 Sep 14;33(7):3944–59. doi: 10.1093/cercor/bhac318 (PMC10068282; doi:10.1093/cercor/bhac318)
Supplement: Supplementary_Material_bhac318 [file supplementary_material_bhac318.zip › Supplementary_Material_bhac318.docx]

**Supplementary Material:**

*
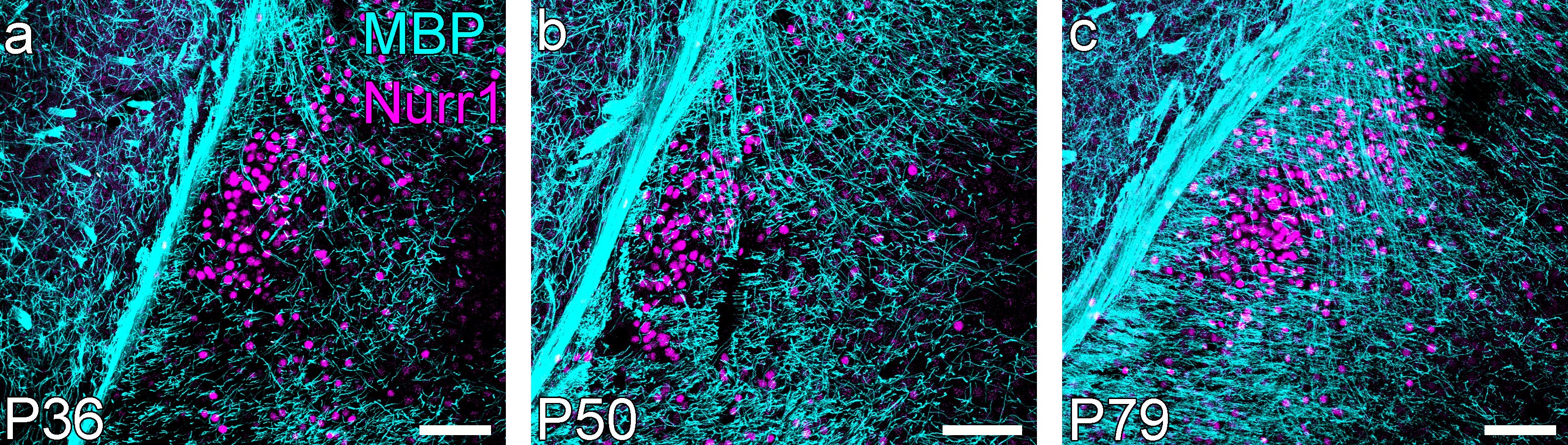
*

***Supplementary Figure 1:*** *At the level of the anterior commissure, the Nurr1+ patch of claustrum cells sits in the centre of the MBP+ ‘bird’s nest’ of myelinated fibres surrounding the claustrum. (a-c) Maximum intensity projection confocal laser scanning microscope images of the claustrum region at different postnatal ages, immunohistochemically stained for Nurr1 (magenta) and MBP (cyan). Scale bars = 100μm*

***
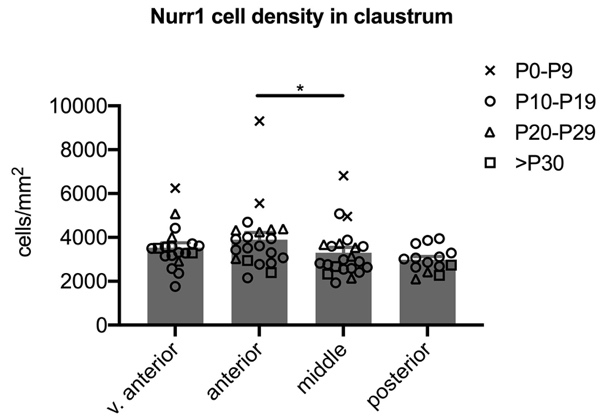
***

***Supplementary Figure 2:*** *Density of Nurr1+ cells in the claustrum is much higher during the first ten days after birth, and stable thereafter. Nurr1+ cell density was quantified in three or four sections for each brain (n=22 brains aged P5-P36). There is a strong effect of age on Nurr1+ cell density in the claustrum (main effects ANOVA F (9, 63) = 16.55, p<0.0001), which is exclusively due to the much higher cell density throughout the claustrum in the youngest (and therefore smallest) two brains (Tukey’s multiple comparison test p<0.01 for all comparisons involving P5 and P8 brains; data represented by ‘x’ in the graph). There is also a significant effect of anterior-posterior position on Nurr1+ cell density within the claustrum (main effects model ANOVA F (3, 63) = 3.701, p=0.0161). Tukey’s multiple comparison test indicates that only the Nurr1+ patch density at the level of the anterior commissure midline-crossing (‘middle claustrum’) is significantly different from the anteriorly adjacent claustrum (Tukey’s multiple comparison test, p=0.0318). * p<0.05*

***
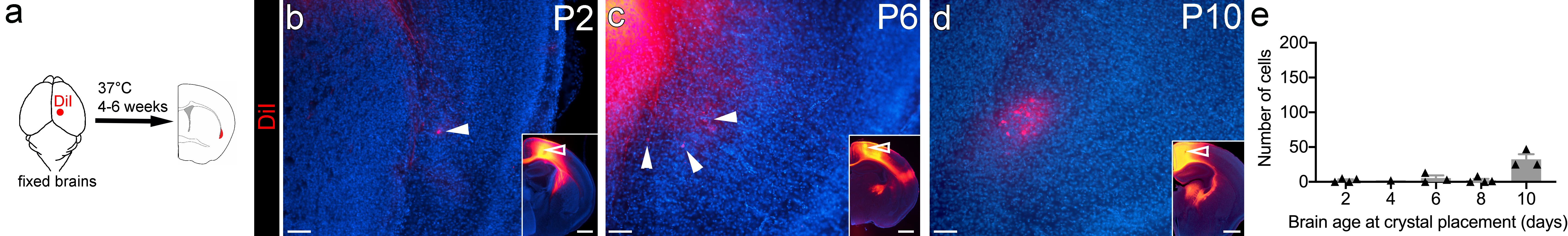
***

***Supplementary Figure 3****: Carbocyanine dye tracing was used in fixed brains to determine the approximate age at which cells in the claustrum and lateral cortex extend axons to RSP (a). (b-d) Epifluorescence images of the claustrum region as delineated by relative cell density of DAPI-stained nuclei (blue), and carbocyanine labelling. The crystal placement site for each brain is shown in the insets, with white open arrowheads pointing at the placement site. A few DiI+ cells were found in the region of the claustrum, even at the youngest ages studied (arrowheads in b, c). By P10, a cluster of retrogradely labelled cells was visible in claustrum (d). (e) Mean±sem of all claustrum cells retrogradely labelled with carbocyanine, counted in every 5^th^ section along the AP extent of the claustrum of a given brain. There was a sharp increase in the number of retrogradely labelled claustrum cells at P10, compared to earlier ages. Scale bars = 100μm and 1mm (insets).*

*
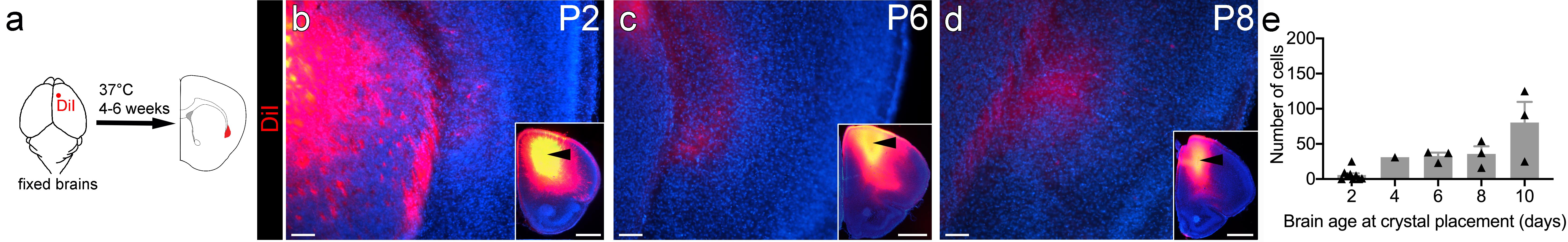
*

***Supplementary Figure 4:*** *Carbocyanine dye tracing was used in fixed brains to determine the approximate age at which cells in the claustrum and lateral cortex extend axons to anterior cingulate cortex (ACA). (b-d) Epifluorescence images of the claustrum region and adjacent lateral cortex as delineated by relative cell density of DAPI-stained nuclei (blue), and carbocyanine labelling. A few cells were found dorsal to the claustrum, and occasionally in the region of the claustrum, even at the youngest ages studied. The crystal placement site for each brain is shown in the insets, with black arrowheads pointing at the placement site. (e) Mean±sem of all retrogradely labelled claustrum cells counted in every 5^th^ section along the AP extent of the claustrum of a given brain. Individual brains shown as circles. There was a steady increase in the number of back-labelled claustrum cells with increasing age when using carbocyanine dye tracing, but this did not reach statistical significance (Spearman’s r=0.9, p=0.0833). Scale bars = 100μm and 1mm (insets).*
